# Electrochemical Determination of Hazardous Herbicide Diuron Using MWCNTs-CS@NGQDs Composite-Modified Glassy Carbon Electrodes

**DOI:** 10.3390/bios13080808

**Published:** 2023-08-11

**Authors:** Jin Zhu, Yi He, Lijun Luo, Libo Li, Tianyan You

**Affiliations:** Key Laboratory of Modern Agricultural Equipment and Technology, Ministry of Education, School of Agricultural Engineering, Jiangsu University, Zhenjiang 212013, China

**Keywords:** diuron, electrochemical sensor, multi-walled carbon nanotubes, nitrogen-doped graphene quantum dot

## Abstract

Diuron (DU) abuse in weed removal and shipping pollution prevention always leads to pesticide residues and poses a risk to human health. In the current research, an innovative electrochemical sensor for DU detection was created using a glassy carbon electrode (GCE) that had been modified with chitosan-encapsulated multi-walled carbon nanotubes (MWCNTs-CS) combined with nitrogen-doped graphene quantum dots (NGQDs). The NGQDs were prepared by high-temperature pyrolysis, and the MWCNTs-CS@NGQDs composite was further prepared by ultrasonic assembly. TEM, UV-Vis, and zeta potential tests were performed to investigate the morphology and properties of MWCNTs-CS@NGQDs. CV and EIS measurements revealed that the assembly of MWCNTs and CS improved the electron transfer ability and effective active area of MWCNTs. Moreover, the introduction of NGQDs further enhanced the detection sensitivity of the designed sensor. The MWCNTs-CS@NGQDs/GCE electrochemical sensor exhibited a wide linear range (0.08~12 μg mL^−1^), a low limit of detection (0.04 μg mL^−1^), and high sensitivity (31.62 μA (μg mL^−1^)^−1^ cm^−2^) for DU detection. Furthermore, the sensor demonstrated good anti-interference performance, reproducibility, and stability. This approach has been effectively employed to determine DU in actual samples, with recovery ranges of 99.4~104% in river water and 90.0~94.6% in soil. The developed electrochemical sensor is a useful tool to detect DU, which is expected to provide a convenient and easy analytical technique for the determination of various bioactive species.

## 1. Introduction

Diuron (DU) is a widely used herbicide for weed removal in croplands for plants such as soybeans, potato, cotton, and citrus fruits [1]. It is also one of the main components of ship anti-fouling paint [2]. Due to the extensive use of DU in the agricultural field and shipping industry, it has been found in rivers and soils in many countries [3]. DU is a toxic, persistent substance with a half-life longer than 300 days [4], and the water pollution caused by DU can lead to cancer, endocrine disorders, and other diseases [5]. Moreover, long-term retention of high doses of DU in soil will eventually enter into the human body through the food chain, posing a potential threat to human health [6]. Therefore, DU has been classified as a priority hazardous substance by the European Commission [7]. The indiscriminate use of this dangerous herbicide has posed imminent risks to human and aquatic life; therefore, it is of great significance to detect DU residue in the soil and the aquatic environment. At present, many analytical methods for DU, including high-performance liquid chromatography–tandem mass spectrometry (HPLC-MS) [8], capillary electrophoresis coupled with electrochemiluminescence (CE-ECL) [9], surface-enhanced Raman spectroscopy (SERS) [10], and fluorescence immunoassay (FL) [11], have been developed. Although these methods exhibit the benefits of high sensitivity and outstanding accuracy, the complicated operation and expensive equipment limit their large-scale application [12,13]. In contrast, the electrochemical technique is a simple, rapid, and cost-effective analytical method which has been used recently to detect DU [14,15].

The electrochemical approach for DU detection is primarily based on the principle that DU itself is an electrochemically active substance and can generate a definite electrochemical signal. However, it is very difficult to obtain a high electrochemical response of DU on a bare glassy carbon electrode (GCE), leading to relatively low sensitivity. Fortunately, the sensitivity can be greatly enhanced by modifying nanomaterials on the GCE surface [16]. In the past few decades, numerous functional nanomaterials, including, carbon nanomaterials [17], noble metal nanoparticles [18], metal–organic frameworks [19], and conductive polymers [20] have been widely used to enhance the analytical performances of electrochemical sensors. In particular, carbon nanomaterials exhibited potential applications due to their exceptional catalytic activity, large surface areas, excellent electrical conductivity, and potent analyte adsorption [21]. Among them, multi-walled carbon nanotubes (MWCNTs) are common one-dimensional carbon nanomaterials with high conductivity and large surface areas [22]. Nonetheless, the poor dispersion, weak adhesion, and poor biocompatibility of MWCNTs hinder their application [23]. Chitosan (CS) is a perfect biopolymer that can be employed to boost MWCNT performance, since it has strong film-forming, adhesion, and biocompatibility properties. Many studies have reported that CS and MWCNTs can form stable composites through non-covalent binding [24]. The amino group on the CS surface can enhance the hydrophilic ability of MWCNTs and further promote the dispersion of MWCNTs in CS aqueous solution, thus greatly improving their electrochemical performances.

In addition, graphene quantum dots (GQDs) are zero-dimensional nanoscale pieces of graphene that possess the properties of both graphene and carbon dots [25]. With abundant structural defects of the surface and edge, good water solubility, and excellent electrocatalytic properties [26], GQDs have received widespread attention in the realm of electrochemistry [27]. For example, Gholivand et al. [28] successfully developed an electrochemical sensor to detect the anticancer drug cisplatin based on graphene quantum dots-thionine (GQDs-Thi)-modified porous glass carbon electrodes. Zhang et al. [29] presented an electrochemical sensor using graphene quantum dots-Pt nanoparticles (GQDs-PtNPs)-modified glass carbon electrodes to detect the antibacterial medicine sulfadimidine. Moreover, previous studies have shown that doping of N atoms can further enhance the electrochemical performances of GQDs [30]. For carbon nanocomposites, research has shown that the inherent benefits of different types of materials may be fully used to achieve enhanced electrochemical performances [31]. Therefore, the integration of MWCNTS-CS and NGQDs is expected to provide better electrochemical performances than that of a single material. To our knowledge, no studies regarding electrochemical sensors which have employed a combination of MWCNTs-CS and NGQDs have been reported for the detection of DU.

**Scheme 1:**
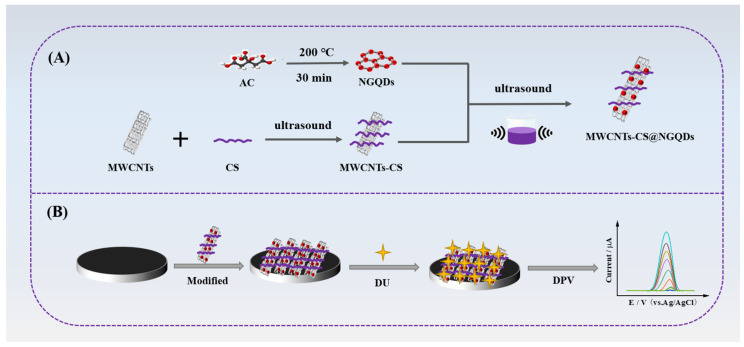
(**A**) Synthetic route of MWCNTs-CS@NGQDs composite and (**B**) the detection process of DU based on the MWCNTs-CS@NGQDs/GCE electrochemical sensor.

In the current work, we developed a novel electrochemical sensor utilizing MWCNTs-CS@NGQDs composite-modified GCE to detect DU. As shown in Scheme 1A, NGQDs were first synthesized by pyrolyzing ammonium citrate (AC) at high temperature. Then, the MWCNTs-CS@NGQDs composite was prepared using the one-step simple ultrasonic self-assembly method based on the electrostatic interaction between MWCNTs-CS and NGQDs. The functional materials were further modified on the surface of GCE to construct an electrochemical sensor to achieve direct and sensitive detection of DU (Scheme 1B). Benefiting from the aforementioned advantages, the MWCNTs-CS@NGQDs/GCE sensor demonstrated high sensitivity, dependable reproducibility, and excellent stability for the determination of DU. Finally, by measuring DU in river water and soil samples, the practical feasibility of the electrochemical sensor was evaluated.

## 2. Experimental Section

### 2.1. Reagents and Instrumentation

Anhydrous ethanol (C₂H₆O), acetonitrile (C₂H_3_N), potassium ferricyanide (K_3_[Fe(CN)_6_]), potassium ferrocyanide (K_4_[Fe(CN)_6_]·3H_2_O), potassium chloride (KCl), Sodium chloridesodium (NaCl), hydroxide (NaOH), ammonium citrate (C_6_H_17_N_3_O_7_), boric acid (95%), acetic acid (95%), phosphoric acid (95%), and chitosan (CS) were purchased from Sinopharm Chemical Reagent Co., Ltd. (Shanghai, China). MWCNTs were acquired from Nanjing Pioneer Nano Technology Co, Ltd. (Nanjing, China). DU was obtained from Aladdin (Shanghai, China). The Milli-Q ultrapure water production equipment provided the water for the experiment. All chemicals and reagents were of analytical grade.

Morphological and structural investigations were conducted using an FEI Tecnai G2 F20 field-emission transmission electron microscope (FEI, Hillsboro, OR, USA). The surface charges of different materials were measured by a zeta-sizer Nano-ZS (Malvern, UK). Ultraviolet-visible (UV-Vis) spectra of different materials were obtained with a UV-3600 plus spectrophotometer (Shimadzu, Kyoto, Japan). FL (fluorescence) spectra were produced by means of an FS5 FL spectrophotometer (Edinburgh Instruments, UK). Electrochemical impedance spectroscopy (EIS) was recorded on a SP-200 electrochemical workstation (Biologic, France). Other electrochemical experiments were carried out on a CHI852D electrochemical workstation (Chenhua Co., Ltd., Shanghai, China).

### 2.2. Preparation of NGQDs

NGQDs were prepared by the simple reflux method at atmospheric pressure according to the methods described in previous literature [32]. Specifically, a three-necked flask containing 2 g AC and 60 mL H_2_O was heated to 200 °C using an oil bath pan. The steam produced at high temperatures was condensed into liquid using a reflux condenser during the experiment. In order to allow for the doping of N atoms into GQDs, a balloon was placed at the outlet of the condenser tube. The balloon had two purposes: the first was to keep the environment closed, and the second was to check that the experiment was carried out at a standard atmospheric pressure. When NGQDs began to develop, the color of solution changed from colorless to light yellow. Finally, the obtained NGQDs were then stored in the refrigerator at 4 °C for future use.

### 2.3. Preparation of the MWCNTs-CS@NGQDs Composite

The MWCNTs-CS@NGQDs composite was prepared by a simple ultrasonic assembly method. Specifically, 2 mg MWCNTs and 4 mg CS were dispersed in 1 mL 2% acetic acid for ultrasonic treatment for 1 h to obtain the MWCNTs-CS composite. At the same time, the as-prepared NGQDs were diluted to a concentration of 12 mg mL^−1^. Finally, the MWCNTs-CS dispersion was mixed with NGQDs for ultrasonic treatment for 1 h to obtain the MWCNTs-CS@NGQDs composite.

### 2.4. Fabrication of the Electrochemical Sensor

Before fabrication, the GCE was firstly polished with alumina slurries powder. Afterward, the electrode was cleaned 3 times for 3 min by sequential ultrasonic treatment (40 kHz, 120 W) with ultrapure water, ethanol, and ultrapure water. Finally, 2.5 μL of the MWCNTs-CS@NGQDs dispersion was deposited onto the clean GCE surface; then, the electrode was placed in an oven at 35 °C for 30 min to allow for the complete evaporation of the solvent.

### 2.5. Electrochemical Measurements

A traditional three-electrode system was arranged for the electrochemical experiments. The working electrode was a GCE (d = 2 mm), the platinum wire electrode was selected as the counter electrode, and Ag/AgCl was utilized as the reference electrode, respectively. Cyclic voltammetry (CV) and electrochemical impedance spectroscopy (EIS) were measured to evaluate the electrochemical performances of the different modified GCEs. The potential range for CV was initially set between −0.2 and 0.6 V, and the scanning rate was set at 0.1 V s^−1^. The initial parameters of EIS were as follows: the signal amplitude was 5 mV and the frequency range was 10~10^6^ Hz. The differential pulse voltammetry (DPV) method was applied to record the electrochemical signal of DU. For detailed measurements, the modified GCE was placed in B-R buffer solution (pH = 2.0) containing DU and accumulated for 180 s to complete the adsorption of DU. The following DPV parameters were used to detect DU: a potential range of 0.95 to 1.35 V, a pulse width of 0.05 s, an impulse amplitude of 0.05 V, and a pulse period of 0.5 s.

### 2.6. Application in Practical Sample Analysis

The river water sample was collected from the Beijing-Hangzhou Grand Canal in Zhenjiang, China, according to the standard methods. The soil was obtained from the Xinjiang region. First, 5 g of crushed soil samples were added into 5 mL of water and 10 mL of acetonitrile, then extracted by shaking for 10 min. Afterwards, 4 g NaCl was introduced to induce the separation of aqueous phase and the organic phase, and then the samples were centrifugated at a high speed of 4000 r/min for 5 min. The soil supernatant and river water samples were filtered through a 0.22 μm filter membrane to obtain the test samples. Finally, the test samples were diluted 20 times with 0.04 M B-R buffer (pH = 2.0). DU at different concentrations was spiked into the diluted solutions, and then the solutions received ultrasonic treatment for 5 min. The proposed electrochemical sensor was used to assess DU residues in the practical samples. Finally, the experimental results were compared with the national standard method, i.e., ultrahigh-performance liquid chromatography–tandem mass spectrometry (UPLC-MS/MS).

## 3. Results and Discussion

### 3.1. Characterization of NGQDs

**Figure 1:**
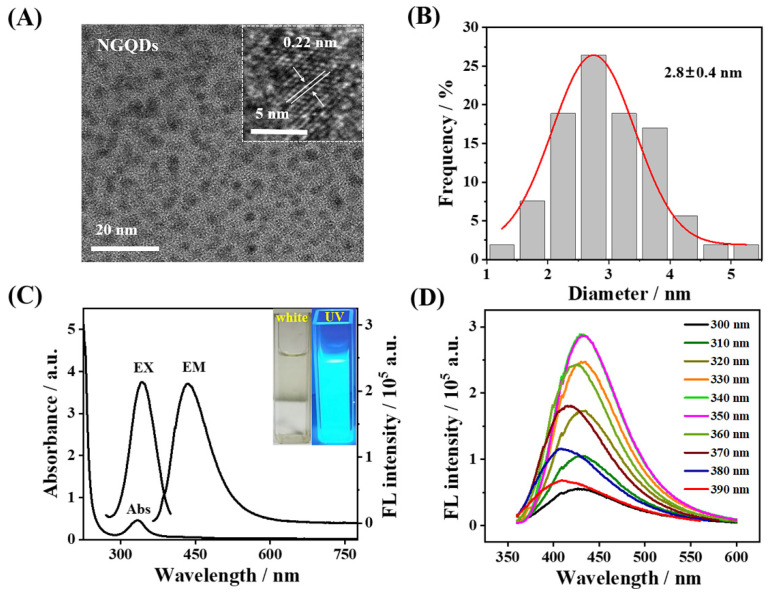
(**A**) TEM image of NGQDs. Inset, HRTEM image of NGQDs. (**B**) Particle size distribution of NGQDs. (**C**) UV-vis absorption spectrum and FL spectra of NGQDs. Inset, white and 365 nm UV light images of NGQDs, respectively. (**D**) Dependence of emission on excitation of the as-prepared NGQDs.

The morphology of NGQDs was characterized using TEM. It can be observed from Figure 1A that the synthesized NGQDs were uniformly distributed. The HRTEM image shows that the lattice spacing of NGQDs was 2.20 Å (Inset of Figure 1A), matching well with the (100) facet of graphitic carbon [33]. The average particle size of the synthesized NGQDs was 2.8 ± 0.4 nm, with a relatively narrow particle size range of 1.7~5.2 nm (Figure 1B). The optical characteristics of NGQDs were determined by UV-vis and FL spectrometry. As can been seen from the UV-vis spectrum, the remarked UV absorption peak of NGQDs at 335 nm can be attributed to the π-π* conjugate structures of aromatic sp^2^ domains [30] in the NGQDs (Figure 1C). The inset of Figure 1C illustrates that the obtained NGQDs solution emitted blue fluorescence when exposed to a 365 nm UV light. The maximum excitation and emission wavelengths for NGQDs were located at 350 nm and 435 nm, respectively (Figure 1C). For the purpose of further investigating the optical performances of NGQDs, the emission wavelengths of NGQDs at different excitation wavelengths were investigated. In carbon-based fluorescence materials, the specific dependence of the emission wavelength and fluorescence intensity on the excitation wavelength is a common phenomenon in the intrinsic state luminescence mechanism [34]. On the contrary, the photoluminescence of NGQDs is independent of excitation (Figure 1D). The position of the emission peak changes little with the constant transition of the excitation wavelength from 300 to 390 nm, demonstrating that the surface state of NGQDs is very uniform [35].

### 3.2. Characterization of MWCNTs-CS@NGQDs Composite

**Figure 2:**
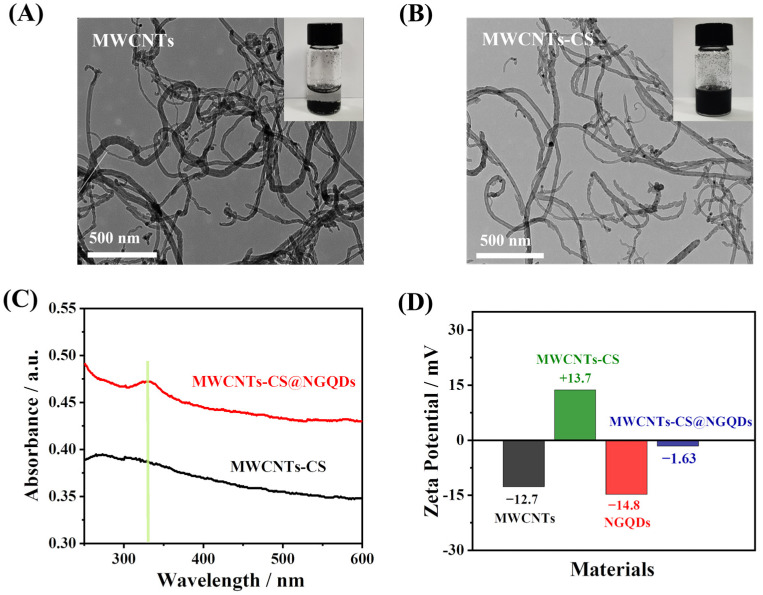
TEM images of (**A**) MWCNTs and (**B**) MWCNTs-CS. Insets, photographs of (**A**) MWCNTs and (**B**) MWCNTs-CS dispersed in water. (**C**) UV-vis absorption spectrum of MWCNTs-CS and MWCNTs-CS@NGQDs. (**D**) Zeta potentials of different materials.

The TEM morphologies of MWCNTs and MWCNTs-CS were firstly studied. Figure 2A shows the TEM image of MWCNTs, from which it can be found that MWCNTs show small bundles or single tubular structures with different, irregular, and interwoven lengths. The material can significantly increase the electroactive area of GCE and effectively enhance the electron transfer ability [36]. However, due to the strong Van der Waals interactions [37], MWCNTs inevitably aggregate in aqueous solution, resulting in poor dispersion (Inset of Figure 2A). Upon the addition of CS, the dispersion of MWCNTs-CS is significantly improved (Inset of Figure 2B), thus increasing the surface area of MWCNTs. Figure 2B shows that the MWCNTs-CS still retain their tubular structures. The tubulose networks work as conductive channels where electrons and ions can transport rapidly. UV-vis spectroscopy was then conducted to characterize the formation of MWCNTs-CS@NGQDs. Figure 2C illustrates that MWCNTs-CS have no significant absorption peaks in the 250~600 nm range. However, upon the addition of NGQDs, the MWCNTs-CS@NGQDs exhibited a clear UV absorption peak at 335 nm, which can be attributed to the π-π* conjugate structures of aromatic sp^2^ domains in the NGQDs. The results indicate the successful combination of NGQDs and MWCNTs-CS. Furthermore, the zeta potential values of different materials were recorded. As shown in Figure 2D, both MWCNTs (−12.7 mV) and NGQDs (−14.8 mV) were negatively charged. Interestingly, when mixing CS with MWCNTs, the zeta potential of MWCNTs changed from −12.7 mV to 13.7 mV. Furthermore, the zeta potential value of MWCNTs-CS@NGQDs was determined as −1.63 mV, suggesting that MWCNTs-CS and NGQDs can indeed be combined together via electrostatic interaction.

### 3.3. Electrochemical Characterization of Different Modified GCE

**Figure 3:**
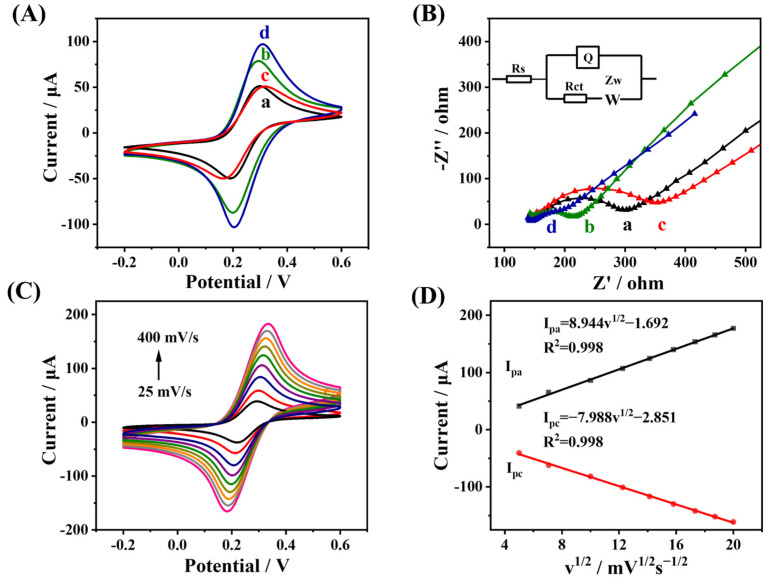
(**A**) CV and (**B**) EIS responses of different modified electrodes, including (a) GCE, (b) MWCNTs-CS/GCE, (c) NGQDs/GCE, and (d) MWCNTs-CS@NGQDs/GCE in 0.1 M KCl containing 5 mM [Fe(CN)_6_]^3−/4−^. (**C**) CV responses of MWCNTs-CS@NGQDs/GCE at various scan rates (25~400 mV s^−1^) in 0.1 M KCl containing 5 mM [Fe(CN)_6_]^3−/4−^. (**D**) Linear curves of redox peak currents versus v^1/2^.

Figure 3A shows the CV curves of GCE, MWCNTs-CS/GCE, NGQDs/GCE, and MWCNTs-CS@NGQDs/GCE. For GCE (curve a), the anodic peak current (I_pa_) value was 57.27 μA and the peak separation potential (ΔE_p_) was 108 mV. For MWCNTs-CS/GCE (curve b), the I_pa_ value was obviously enhanced (85.98 μA), and ΔE_p_ reduced to 99 mV due to the excellent electrical conductivity of MWCNTs-CS. For NGQDs/GCE (curve c), the I_pa_ value was slightly decreased (53.58 μA) and ΔE_p_ increased from 108 mV to 125 mV, which may be due to the electrostatic repulsion between the negatively charged carboxyl group in NGQDs and the negatively charged [Fe(CN)_6_]^3−/4−^. As expected, the MWCNTs-CS@NGQDs/GCE showed a significantly enhanced I_pa_ value (98.52 μA) and a reduced ΔE_p_ value (96 mV) when compared to the MWCNTs-CS/GCE and NGQDs/GCE (curve d), indicating that the MWCNTs-CS@NGQDs/GCE has better electron transfer efficiency, which may be related to the outstanding electron transport capability and large specific surface area of MWCNTs-CS@NGQDs.

The EIS experiment was also conducted to investigate the electrical and electron transport properties at the interface electrode/solution. The inset of Figure 3B shows the equivalent circuit suitable for the experimental results. Embedded Randles equivalent circuits include Warburg impedance (Z_w_), charge transfer impedance (R_ct_), solution impedance (R_s_), and double layer capacitance value (Q). In a Nyquist diagram, the diameter of a semicircle was utilized to calculate the R_ct_ of various sensors. Figure 3B shows the Nyquist plots of GCE, MWCNTs-CS/GCE, NGQDs/GCE, and MWCNTs-CS@NGQDs, respectively. When modified with MWCNTs-CS, MWCNTs-CS/GCE had a lower R_ct_ value (72.6 Ω, curve b) than bare GCE. (140.5 Ω, curve a). But for NGQDs/GCE, the value of R_ct_ was 205.2 Ω (curve c). For MWCNTs-CS/NGQD/GCE, the R_ct_ value was the lowest (50.7 Ω, curve d), indicating that MWCNTs-CS@NGQDs/GCE possessed the strongest electron transfer capacity.

The CV responses of the MWCNTs-CS@NGQDs/GCE at different scan rates from 25 to 400 mV s^−1^ were then recorded (Figure 3C). The square roots of the scan rate (v^1/2^) are linearly correlated with the I_pa_ and cathode peak current values (I_pc_) (Figure 3D), suggesting that the electrochemical reaction of [Fe(CN)_6_]^3−/4−^ is controlled by diffusion [16]. The Randles–Sevcik equation was used to determine the electrochemical active surface area of several modified electrodes [38]:

I_p_ = (2.69 × 10^5^)n^3/2^D^1/2^AC v^1/2^ (1)

where D is the diffusion coefficient of potassium ferricyanide (equal to 7.6 × 10^−6^ cm^2^ s^−1^), C is the concentration of the redox probe (M), v is the scan rate (mV s^−1^), I_p_ is the redox peak current, n is the number of electrons involved in the redox reaction, A is the electrochemical active surface area (cm^2^), and n is the number of electrons involved in the redox reaction. The electroactive surface areas of bare GCE, MWCNTs-CS/GCE, NGQDs/GCE, and MWCNTs-CS@NGQDs/GCE were calculated to be 0.052 cm^2^, 0.082 cm^2^, 0.045 cm^2^, and 0.095 cm^2^, respectively, indicating that MWCNTs-CS@NGQDs/GCE possessed the largest active area of any of the modified electrodes and was able to detect DU more effectively.

### 3.4. DPV Responses of DU at Different Modified GCE and Oxidation Mechanism of DU

**Figure 4:**
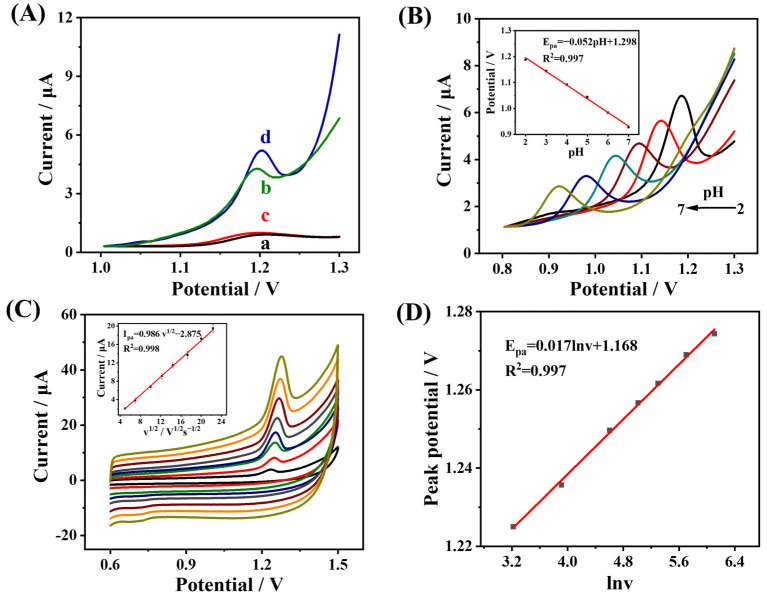
(**A**) DPVs curves of DU in B-R buffer (pH 2.0) at (a) GCE, (b) MWCNTs-CS/GCE, (c) NGQDs/GCE, and (d) MWCNTs-CS@NGQDs/GCE, respectively. (**B**) DPVs responses of MWCNTs-CS@NGQDs/GCE in B-R buffer at various pH values. Inset, the dependence of the E_pa_ on the pH. (**C**) CVs responses to DU under different scanning rates. Inset, the corresponding plot of I_pa_ vs. v^1/2^. (**D**) The corresponding plot of lnv vs. E_pa_.

DPV responses of different modified electrodes in 0.04 M B-R buffer solution containing 4 μg mL^−1^ DU were investigated (Figure 4A). For bare GCE (curve a) and NGQDs/GCE (curve c), a certain degree of electrochemical responses of DU was observed, but the current intensity was not sufficient to meet the detection requirement. In contrast, distinct oxidation peaks were observed at MWCNTs-CS/GCE (curve b) and MWCNTs-CS@NGQDs/GCE (curve d). Two reasons can account for these results: on the one hand, the participation of CS enriches the functional groups of MWCNTs, which is conducive to the adsorption capacity of DU on MWCNTs-CS/GCE [39]; while on the other hand, the assembly of MWCNTs-CS and NGQDs can produce more reactive active sites [40] to further improve the current response to DU. Therefore, MWCNTs-CS@NGQDs/GCE exhibited the best current response of DU. 

In order to explore the oxidation mechanism of DU, the effects of pH value as well as scan rates on the anode peak potential (E_pa_) and oxidation current (I_pa_) of DU were studied. As can been observed from Figure 4B, with the increases in pH value, the I_pa_ value gradually decreased, which was the expected behavior of DU according to previous reports [1]. This can be interpreted as the acidic medium protonating the amino group in DU more easily [41], thus promoting the electrochemical oxidation process of DU. Furthermore, it can be observed that the oxidation process of the analyte depended on the pH value. As the pH increased, the DU oxidation peak potential shifted negatively, suggesting that protons may be involved in the electrochemical oxidation process of DU [42]. The inset of Figure 4B depicts the linear relationship between the peak potential and the pH value of DU. The linear equation is E_pa_(V) = −0.052 pH + 1.298, with an R^2^ of 0.997. The slope in the equation, which is extremely near to the theoretical value for the transfer of an equal number of protons and electrons (−59 mV/pH), indicates that the DU oxidation reaction involves an equal amount of proton and electron transfer [43].

**Figure 5:**
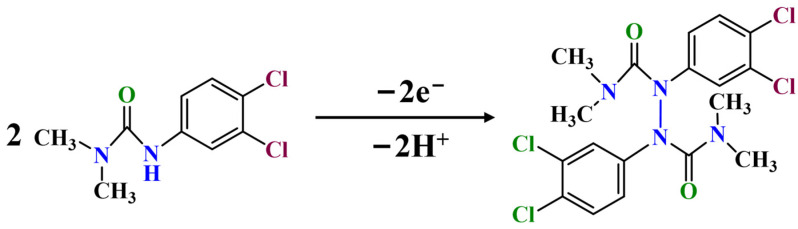
The electrochemical oxidation mechanism of DU.

Next, the CV curves under various scan rates (25~500 mV s^−1^) in B-R buffer containing 4 μg mL^−1^ DU were recorded. As shown in Figure 4C, the electrochemical oxidation of DU appears to be a typical diffusion-controlled process [44] and the linear equation is written as: I_pa_(μA) = −2.875 + 0.986 v^1/2^ with R^2^ of 0.998 (Inset of Figure 4C). Additionally, E_pa_(V) has a linear relationship with lnv, with the following linear equation: E_pa_(V) = 1.168 + 0.017 lnv with R^2^ of 0.997 (Figure 4D). The Bulter–Volmer equation is as follows [45]:

(2) $$E_{p}={\;E}^{0}+\frac{\mathrm{RT}}{\alpha \mathrm{nF}}\ln\frac{\mathrm{RT}k^{0}}{\alpha \mathrm{nF}}+\frac{\mathrm{RT}}{\;\alpha \mathrm{nF}}\mathrm{lnv}$$

where R, F, T, and v represent the gas constant (R = 8.314 J mol^−1^ K^−1^), faraday constant (F = 96485 C mol^−1^), surrounding temperature (298 K), and scan rate (mV s^−1^), respectively. The slope of the equation can be represented as RT/αnF. The value can be assumed to be 0.5 for the irreversible oxidation reaction [21]. The n value was calculated as 2.04, suggesting that a two-proton and two-electron transfer process is involved in the DU reaction mechanism in the MWCNTs-CS@NGQDs/GCE sensor. The above experimental results indicate that the oxidation process of DU may be caused by N-H bond breaking to form N-N bonds involving 2H^+^/2e^−^ transfer [5], as suggested in Figure 5.

### 3.5. Determination of DU by the Developed Electrochemical Sensor

**Figure 6:**
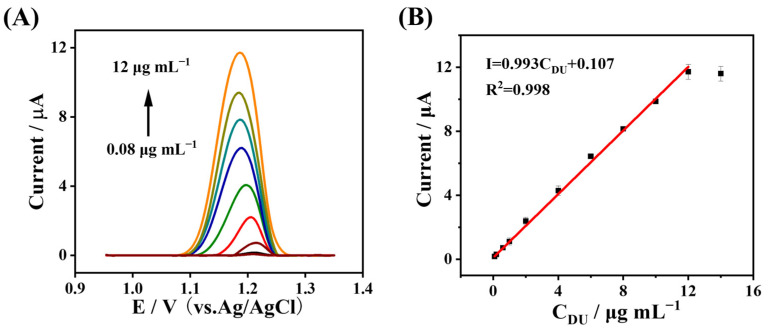
(**A**) DPV response of DU at different concentrations. (**B**) The corresponding calibration plots of the DU concentration vs. the peak current.

Under the optimal experimental conditions (Figure S1), the peak current response of DU in the fabricated MWCNTs-CS@NGQDs/GCE sensor was explored via the DPV approach. As displayed in Figure 6A, the oxidation current increased gradually as the DU concentration increased. In the range from 0.08 to 12 μg mL^−1^, the linear equation for the association between the current responses of DU and its concentration was I(μA) = 0.993C (μg mL^−1^) + 0.107 (R^2^ = 0.998) (Figure 6B). Moreover, the limit of detection (LOD) of DU by the MWCNTs-CS@NGQDs/GCE electrochemical sensor was calculated to be 0.04 μg mL^−1^. The sensitivity from the slope of DU detection was calculated as 31.62 μA (μg mL^−1^)^−1^ cm^−2^. Table 1 lists the results of the analytical performances of other reported methods compared with this DU electrochemical sensor. Compared with other methods, such as HPLC-MS [8], CE-ECL [9], and FL [11], although the analytical performance of this method is inferior, the developed electrochemical sensor is simple and low-cost in terms of the fabrication process. In addition, compared to some reported electrochemical sensors [5,41,46,47,48], the developed electrochemical sensor exhibits certain advantages, i.e., a lower LOD and a wider linear range. This is because the MWCNTs-CS@NGQDs composite provides a tubular conducting network structure, a fast electron/ion transport capability, and a larger specific surface area, thus resulting in good DU analytical performances [49,50].

### 3.6. Anti-Interference Performance, Reproducibility, and Stability Investigation

**Figure 7:**
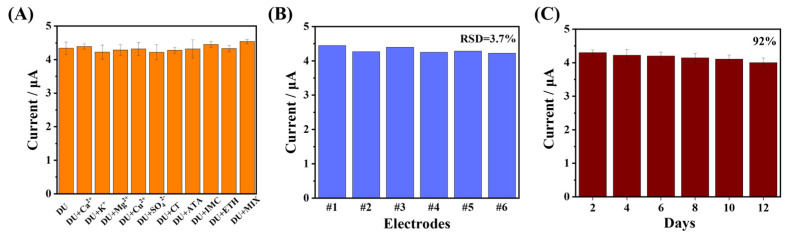
(**A**) Anti-interference ability of the MWCNTs-CS@NGQDs/GCE sensor in the presence of different interferences. (**B**) Reproducibility of six modified electrodes. (**C**) Stability measurement of the developed sensor across 12 days.

The anti-interference performance of the developed sensor was also studied (Figure 7A). The interfering ions and pesticides that may coexist with DU, such as Ca^2+^, Mg^2+^, K^+^, Cu^2+^, SO_4_^2−^, Cl^−^, acetamidine, imidacloprid, and ethephon, were investigated. The concentration of the selected interfering ion was 100 times higher than that of DU, while the concentrations of other pesticides were the same as that of DU. The current changes in the presence of DU were used to evaluate the effect of interfering substances on DU. These interfering substances have little effect on DU detection, indicating that the developed sensor has good anti-interference ability. Six separate modified electrodes were used to detect the same concentration of DU solution. From Figure 7B, it can be observed that the current values of the six electrodes were not significantly different and the relative standard deviation (RSD) was 3.7%, showing that the sensor has good repeatability. Moreover, the storage stability of the modified electrodes was investigated at room temperature for 12 days. With the extension of time, the peak current decreased slightly, and the current decreased to 92% of the initial current on the 12th day (Figure 7C). The results were also within the acceptable range, showing that the proposed sensor possesses good storage stability.

### 3.7. Practical Application 

To assess the applicability of the developed sensor in a practical sample, the as-fabricated electrochemical sensor was applied to detect DU in river water and soil samples by means of the standard addition method. In addition, UPLC-MS/MS was conducted to verify the accuracy of the proposed approach. To reduce matrix interference, the prepared samples were diluted 20 times with 0.04 M B-R buffer initially. Afterward, the samples mentioned above were spiked with the standard solution of DU in three different concentrations. As exhibited in Table 2, the recovery ranges of river water were 99.4~104%, the RSD values were below 5.8%, and the recovery ranges of the soil sample were 90.0~94.6%, with RSD values below 4.8%. By comparison, the results of electrochemical detection were almost consistent with the UPLC-MS/MS results, indicating that the MWCNTs-CS@NGQDs/GCE electrochemical sensor has good applicability for DU detection in practical applications.

## 4. Conclusions

In summary, a novel electrochemical sensor using a MWCNTs-CS@NGQDs-modified GCE was successfully developed to determine DU in river water and soil. The sensor was easily designed, and the fabricated sensor showed an excellent analytical performance regarding DU. This is mainly attributed to the high electron transfer capacity of MWCNTs-CS, as well as abundant active sites of NGQDs. The combination of these endows the sensor with excellent detection performance for DU, including a wide linear range, a low detection limit, and high sensitivity. Additionally, the developed sensor also showed good anti-interference, reproducibility, and stability. This technique has been successfully used to detect DU in practical samples. Meanwhile, the developed electrochemical sensor is expected to provide a rapid, economical, and accurate strategy for the detection of other pesticide residues, presenting potential for its application in environmental analysis. Furthermore, this approach provides a path for the design and synthesis of electrochemical sensing materials, which is expected to broaden the research field of material chemistry.

## Figures and Tables

**Table 1:** Comparison of different methods for DU detection.

Method	Linear Range (μg mL^−1^)	LOD (μg mL^−1^)	Reference
HPLC-MS	-	1.6 × 10^−3^	[8]
CE-ECL	1.0 × 10^−4^–20	1.0 × 10^−5^	[10]
FL	1.0 × 10^−3^−10	1.0 × 10^−5^	[11]
rGO/GCE	1.17−11.7	0.08	[5]
Sa(DODAB)/poly(ARS) /GCE	0.2−3.26	0.11	[48]
GO-MWCNTs/GCE	2.10−85.5	0.15	[49]
PtNPs-CS/GCE	0.04−1.00	0.02	[41]
Hemin-NiOPc-GO/CPE	1.17−32.6	0.39	[50]
MWCNTs-CS@NGQDs/GCE	0.08−12.0	0.04	This work

**Table 2:** Detection of DU in actual samples using the designed sensor (n = 3) and the UPLC-MS/MS standard method.

Samples	Added (μg mL^−1^)	Developed Sensor			UPLC-MS/MS	
		Detected (μg mL^−1^)	Recovery (%)	RSD (%)	Detected (μg mL^−1^)	Recovery (%)
River water	0	-	-	-	0.03	-
	1.00	1.04	104.0	4.5	0.88	88.0
	5.00	5.12	103.7	2.6	4.22	84.6
	10.0	9.94	99.4	5.8	9.11	91.1
Soil	0	-	-	-	0.03	-
	1.00	0.9	90.0	4.5	0.89	89.0
	5.00	4.73	94.6	4.2	3.80	76.0
	10.0	9.06	90.6	4.8	9.32	93.2

“-”, undetected.

## Data Availability

Not applicable.

## Supplementary Materials

The following supporting information can be downloaded at: https://www.mdpi.com/article/10.3390/bios13080808/s1, Figure S1: Optimization of experimental condition.
