# Supplementary material for: Electrochemical Determination of Hazardous Herbicide Diuron Using MWCNTs-CS@NGQDs Composite-Modified Glassy Carbon Electrodes
Source: Biosensors (Basel). 2023 Aug 11;13(8):808. doi: 10.3390/bios13080808 (PMC10452230; doi:10.3390/bios13080808)
Supplement: Supplementary file 1 [file biosensors-13-00808-s001.zip › biosensors-2469862-supplementary.pdf]

biosensors

MDPI

Supplementary Materials

Electrochemical Determination of Hazardous Herbicide Diuron using MWCNTs-CS@NGQDs Composite-Modified Glassy Carbon Electrodes

Jin Zhu, Yi He, Lijun Luo, Libo Li*, and Tianyan You*

Key Laboratory of Modern Agricultural Equipment and Technology, Ministry of Education; School of Agricultural Engineering, Jiangsu University, Zhenjiang, Jiangsu 212013, China
* Correspondence: E-mail: lbli@ujs.edu.cn (L. Li); youty@ujs.edu.cn (T. You); Tel.: +86-511-88982992

Optimization of Experimental Condition

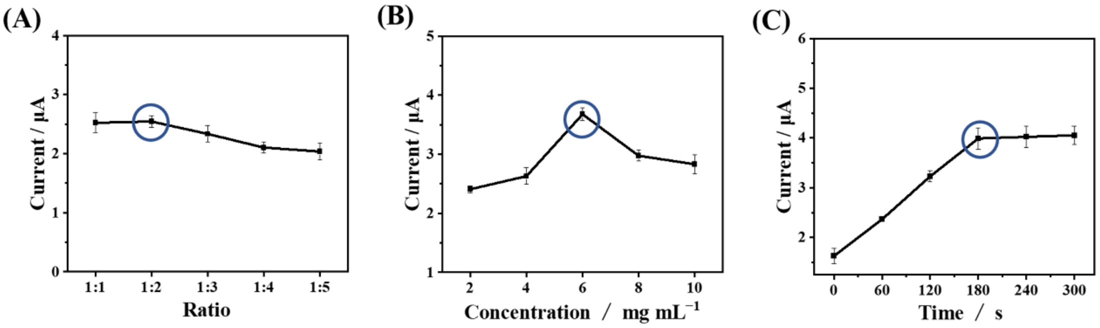

Figure. S1. Condition optimization: (A) the mass ratio of MWCNTs to CS, (B) the concentration of NGQDs, and (C) adsorption time.

In order to obtain the strongest current response of DU, the different mass ratio of MWCNTs to CS was studied (Figure. S1A). The maximum current value was in the ratio of 1:2. With the increase in CS, the current response of DU worsened. Although moderate CS could improve the dispersion of MWCNTs and enhance the performance of MWCNTs, excessive CS was not conducive to the oxidation of DU. This is possibly because CS itself is a low-conductivity substance, hindering the electron transfer of DU.

The NGQDs concentration was another important factor affecting the current response of DU. As shown in Figure. S1B, as the concentration of NGQDs increased, the current response of DU first increased and then decreased. It is worth noting that the peak current reached its maximum when the concentration of NGQDs was 6 mg mL^{-1}. Therefore, 6 mg mL^{-1} was used as the optimal concentration.

Considering the influence of nanomaterials on DU enrichment, the static adsorption time was explored as an important factor for optimization, and the relationship between the peak current and the adsorption time in the range of 0~300 s was measured (Figure. S1C). With the extension of adsorption time from 0~180 s, the peak current continued to increase. When the time was extended, the peak current was almost unchanged, indicating that the adsorption of DU on the electrode had reached saturation. Therefore, 180 s was used as the optimal adsorption time for follow-up experiments.
